# A Quantitative Systems Pharmacology Model of Liver Lipid Metabolism for Investigation of Non-Alcoholic Fatty Liver Disease

**DOI:** 10.3389/fphar.2022.910789

**Published:** 2022-07-19

**Authors:** Theodore R. Rieger, Richard J. Allen, Cynthia J. Musante

**Affiliations:** Quantitative Systems Pharmacology, Early Clinical Development, Pfizer Inc, Cambridge, MA, United States

**Keywords:** QSP, NAFLD, liver, mathematical modeling, steatosis, triglyceride, metabolism

## Abstract

Non-alcoholic fatty liver disease is a metabolic and inflammatory disease that afflicts many people worldwide and presently has few treatment options. To enhance the preclinical to clinical translation and the design of early clinical trials for novel therapeutics, we developed a Quantitative Systems Pharmacology model of human hepatocyte lipid metabolism. The intended application of the model is for simulating anti-steatotic therapies for reversing fatty liver. We parameterized the model using literature data from humans with both normal and elevated liver fat. We assessed that the model construct was sufficient to generate a virtual population of NAFLD patients that matched relevant statistics of a published clinical cohort, and then validated the model response to treatment by simulating pioglitazone and diet intervention in the virtual population. Finally, a sensitivity analysis was performed to determine the best points of intervention for reducing hepatic steatosis. Analysis of the model suggests the most potent method for reducing hepatic steatosis is by limiting non-esterified fatty acid flux from the adipose to the liver.

## 1 Introduction

Non-alcoholic fatty liver disease (NAFLD) is a progressive disorder of the liver that may affect more than 25% of the worldwide population (Younossi et al., 2016; Mitra et al., 2020). While the more advanced stages of the disease (non-alcoholic steatohepatitis, NASH) are notable for inflammation and fibrosis of the liver, the early stages usually begin with steatosis, the accumulation of triglyceride in the hepatocytes (Parthasarathy et al., 2020). The exact molecular mechanism(s) that cause progression of the disease from simple steatosis to the more advanced forms are unknown; however, lipotoxicity from excess lipids inducing an inflammatory response is often postulated as an important driver (Marra and Svegliati-Baroni, 2018). For this reason, pharmacological intervention in the early stages of the disease, i.e., preventing and reversing the accumulation of lipids in the liver, is an attractive therapeutic hypothesis (Calle et al., 2021). Many questions arise in the pre-clinical stages of NAFLD drug development related to the translation of efficacy and safety assessments from animal models to humans. Common concerns include: is the preclinical model representative of the pathological state in humans? Do differences in eating patterns between rodents and humans affect our conclusions? Are there points of pathway intervention in humans that will have more “horsepower” than others for reducing steatosis? Mathematical models, based on human physiology and including mechanisms of disease, can supplement the knowledge we gain from pre-clinical *in vitro* and *in vivo* models to address some of these questions early in drug development.

Quantitative Systems Pharmacology (QSP) models are mechanistic models that utilize our prior knowledge of the biological system, disease mechanisms, and response to treatment informed by data from various sources. QSP models have previously been applied to many physiological systems including COVID-19 (Dai et al., 2021). Notably for understanding NAFLD, there are several prior QSP models that successfully simulate aspects of liver metabolism (Allen and Musante 2018; Noorman et al., 2019; Holzhutter and Berndt 2021; Siler 2022).

Here we developed and analyzed a new model of liver lipid metabolism. In compliment to prior QSP models, we focused the model on simulating therapies for reducing hepatic steatosis in patients with early NAFLD. We focused on developing a “fit-for-purpose” QSP model to aid our goal of developing a model that is mechanistic but highly computationally efficient for simulating early phase clinical trials in NAFLD patients and developing large virtual populations. We demonstrate that the model successfully produces a virtual population with the statistics of a clinical population at steady state and appropriately responds to pioglitazone treatment or dietary intervention. Finally, we use the model and virtual population to perform a sensitivity analysis to identify the pathways that are the most promising intervention points for reducing steatosis as potential monotherapies.

## 2 Methods

### 2.1 Biological Scope of the Model

**FIGURE 1:**
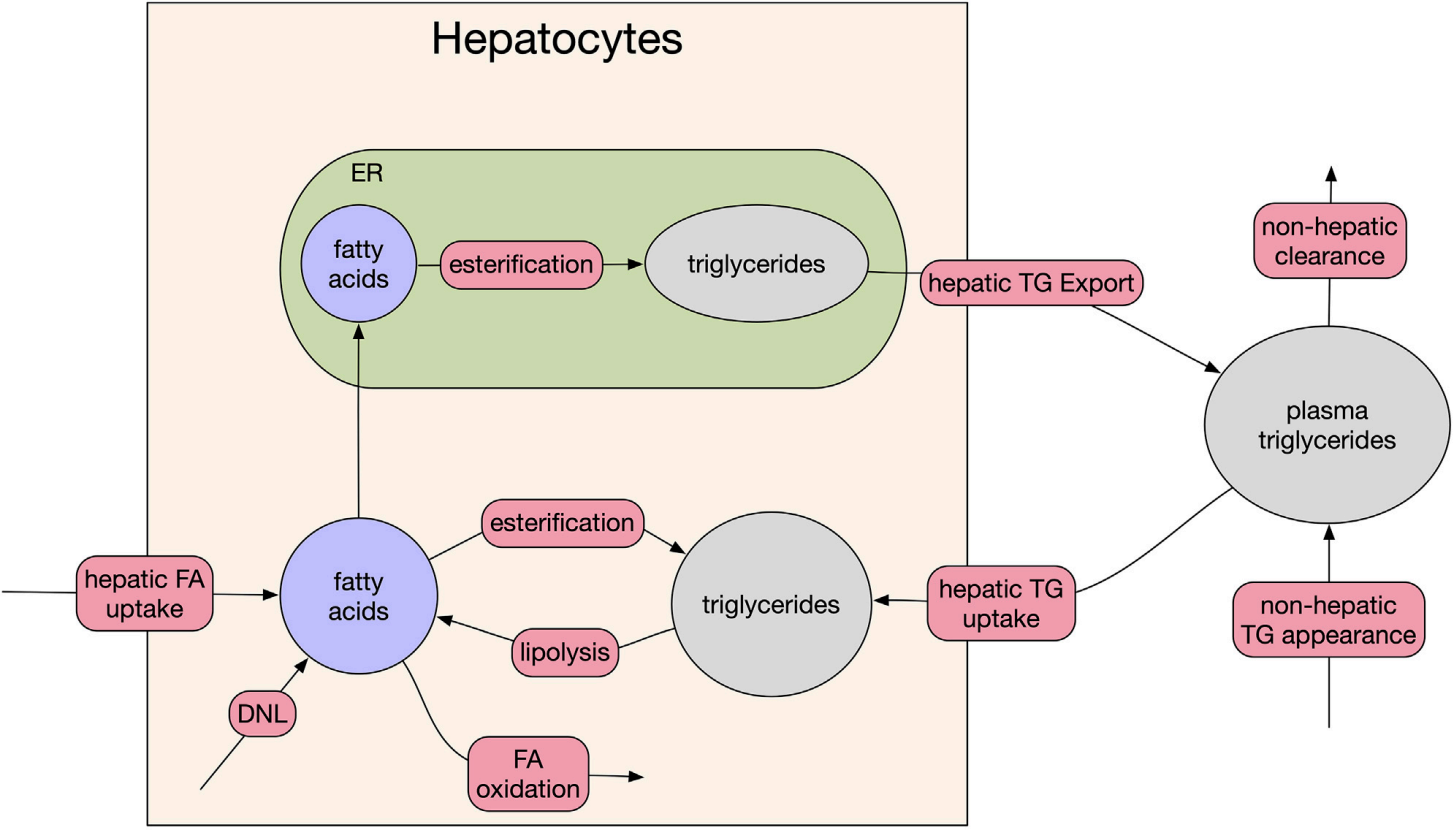
Model schematic. The three compartments of the model are: plasma, hepatocyte cytosol, and hepatocyte endoplasmic reticulum (ER). Red bubbles on arrows represent fluxes of the model, filled ellipses are the five species of the model. For clarity, feedback between DNL and fatty acid (beta) oxidation and cytosolic fatty acid levels and FA uptake are omitted (see source code for full equations).

The model consists of three biological compartments and five dynamic species focused on fatty acids and lipid metabolism (Figure 1). The species are hepatocyte cytosolic fatty acids, hepatocyte cytosolic triglyceride, hepatocyte endoplasmic reticulum (ER) fatty acids, hepatocyte ER triglycerides, and plasma triglycerides. In the model, plasma triglycerides are a lumped representation of all forms of circulating triglycerides including very-low-density lipoprotein (VLDL) synthesized in the liver and chylomicrons from intestinal enterocytes, as well as smaller diameter species such as high-density lipoproteins and low-density lipoproteins.

Since the primary dynamics of interest are the liver triglyceride pools, which do not change appreciably on an hourly basis, several simplifying assumptions were made:

1) The fluxes of the model represent the 24-h average flux (vs. an hourly dynamic). This choice is due to the sparsity of dynamic liver fat data in the literature, usually only being reported pre-/post-treatment after a time span of weeks to month.
2) All inputs to the model are constant (e.g., fatty acid flux from adipose tissue to liver), unless explicitly modified by therapy. Like (1) this choice reflects the sparsity of dynamic data vs. time in clinical studies. Fat mass is usually reported at pre-/post-treatment.
3) No explicit modeling of dynamics such as receptor cycling. As above for (1) and (2), this reflects a time-resolution of available pre-clinical and clinical data. Adding faster dynamics would come at considerable computational cost for no additional certainty in prediction.
4) The mitochondria of hepatocytes will oxidize available fatty acids at a rate determined by the basal metabolic rate of the liver and there is no feedback from carbohydrate content of the hepatocytes.

The focus of the modeling on describing 24-h average fluxes reflects both the lack of data on the minute to hour timescale and the modeling goal of describing clinical trial data points collected weeks apart.

### 2.2 Implementation

We converted the conceptual model (Figure 1) into a system of five non-linear ordinary differential equations. By default, we assumed the kinetics of the model were non-saturating mass-action kinetics. We used saturating kinetics (i.e., Michaelis-Menten type) when we had explicit information for enzymatic constants (e.g., VLDL release from the liver). To reflect some known homeostatic feedback mechanisms, we added two feedback loops, which were free parameters in plausible patient fitting (Sections 2.3, 2.4). First, we added feedback between the rate of *de novo* lipogenesis (DNL) and fatty acid oxidation (McGarry et al., 1977; McGarry et al., 1978). Second, we added feedback between the concentration of cytosolic fatty acids and the uptake rate to reflect homeostatic mechanisms potentially exerted by sterol response element binding protein 1 (SREBP1c), (Ferre and Foufelle 2010).

We implemented the model using the Julia Language, v1.7 (Bezanson et al., 2017). All source code is available online (Rieger et al., 2022).

### 2.3 Base Parameterization

The model contains 22 parameters including basal concentrations and excluding parameters related to therapies. Using published data and steady state constraints on all the pools of the model, we determined a basal value for 20 parameters. The remaining two parameters relate to how the system adapts to dynamic changes from baseline and were allowed to vary across the Virtual Population (Section 2.5).

For creating plausible patients, we established a plausible range for each parameter (log) centered around the pre-determined basal value described above. Absent specific published data, we set the standard deviation of each parameter such that 90% of values are between 0.25 × and 4 × the baseline. The full set of model parameters, their plausible ranges and the derivation of each value is included with the source code (Rieger et al., 2022). The baseline parameters are in Supplementary Table S1, as well as the included supplementary Microsoft Excel sheet: *parameters.xlsx* with the source code. The algebraic derivations of many of the model parameters, using literature or steady state constraints are included in the supplementary Pluto notebook: *derived_parameters.jl.*

### 2.4 NAFLD Virtual Population.

We based our selection of a virtual population on our previously published methodology using Metropolis-Hastings and acceptance-rejection sampling (Rieger and Musante 2016; Rieger et al., 2018). We used the baseline parameterization of Supplementary Table S1 and our estimated variability for the parameters as a joint log-normal distribution. For our naïve search, we assumed the covariance matrix to be diagonal.

The two observables of interest for creating a virtual population were liver fat and plasma triglycerides. We wished to create a general-purpose population that could be sub-selected to make many different patient populations (e.g., healthy normal volunteers, steatotic, hypertriglycerdemic). We digitized the individual subject data shown in Kotronen et al. (2007). (Kotronen and Yki-Jarvinen, 2008), which contained simultaneous measurements of liver fat % and fasting serum triglycerides. Based on the digitized data, we fitted a joint log-normal distribution for the desired statistics of our virtual population. To avoid issues with having to truncate the observable distributions or our parameter distributions, we fitted the full distributions of Kotronen et al. (2007) including both healthy individuals, hyperlipidemics (elevated triglycerides), simple NAFLD, and NAFLD with hyperlipidemia. We allowed the Metropolis-Hastings algorithm to proceed until the algorithm accepted a pre-determined (500,000) number of plausible patients. We then selected our final virtual population using acceptance-rejection sampling (leaving 1,900 virtual patients). Since the data set was for the general population, our final virtual population included both subjects with normal (<5% liver fat) and high liver fat (>5%). We sub-selected all virtual patients with liver fat > 5% as our NAFLD cohort (900) (Chalasani et al., 2018). Due to the stochastic nature of the acceptance-rejection sampling, the final population size varied ± 5% between runs, but the variance of the virtual population selection was found to have no significant effect on the results or conclusions, thus we present the results as a single virtual population run of the model.

### 2.5 Simulation of Pioglitazone Therapy

Pioglitazone is a PPARγ agonist approved for the treatment of type 2 diabetes. While expression of PPARγ has been shown in several tissues of humans, it is highly enriched in adipocytes (Fajas et al., 1997), we assumed the primary pharmacodynamic effect of pioglitazone therapy in humans is insulin-sensitization of adipose tissue (Derosa et al., 2009; Gastaldelli et al., 2009). Thus, the pharmacodynamic effect of pioglitazone therapy was implemented as a reduction in non-esterified fatty acids (NEFAs) released from adipose to liver. For simulation and validation of our model, we selected a study by Belfort et al. (Belfort et al., 2006), which included 24 weeks of pioglitazone or placebo treatment in a randomized cohort of patients (26/group, 45 mg QD) with NAFLD (see Belfort et al. for additional details on the study population and protocol). We estimated the 24-h mean change in NEFAs as the averaged the percent changes observed on the meal challenge and at fasting, representing roughly half the day is spent in the prandial/postprandial period (−28%, Supplementary Material S1). We implemented the effect of pioglitazone in the model as a step change to the uptake flux of NEFAs.

### 2.6 Simulation of Dietary Intervention

We represented the effects of sustained dietary intervention (i.e., reduced food intake) through three simultaneous changes in the model fluxes:

1) Reduced chylomicron influx to the model, representing the reduction in fat consumption.
2) Reduced DNL flux, representing reduced carbohydrate consumption.
3) Reduced NEFA flux to the liver, representing reduced adipose mass.

Importantly, for the purpose of model validation, we wanted to fix these three effects and compare the model predictions to an observed percent change in liver fat. We chose a 26-weeks weight loss study by Haufe et al. (2013). in NAFLD patients (50 patients) for testing our model, see original source for additional details on study protocol and demographics. We converted weight loss into a percentage change in food intake using a published body weight macronutrient model (Hall, 2010). Using the Hall model, we determined that a mean reduction caloric intake of 20% was necessary to achieve the same mean weight loss as Haufe et al. (2013) over 6 months. We fixed the effect on chylomicrons at -20% based on the food intake calculation. Similarly, for DNL flux we used the relationship measured in Schwarz et al. (1995). to calculate a 20% reduction in food intake will reduce DNL by 55% on average. Finally, the NEFA flux was based on the measured change in adipose mass in Haufe et al. (2013) scaled by the 2/3rd factor used by the Hall model for calculating lipolysis rates. Taking these factors together, we reduced NEFA flux by 11% (Supplementary Material for additional details).

### 2.7 Sensitivity Analysis.

Four potential therapeutic strategies were tested:

1) Inhibition of DNL.
2) Inhibition of NEFA uptake to liver.
3) Inhibition of Esterification of Triglycerides in Hepatocytes
4) Activation of VLDL synthesis, increasing triglycerides export from the liver

For each of these, we assumed constant, 24-h inhibition or activation (for VLDL synthesis). We swept each corresponding parameter from 0% → 95% inhibition and simulated our cohort of NAFLD patients for 26 weeks. For activation we swept the inverse of the inhibition (1 × → 20 × the baseline parameter value). The output comparator was the percentage change in liver fat from baseline value.

## 3 Results

### 3.1 Virtual Patient Creation

**FIGURE 2:**
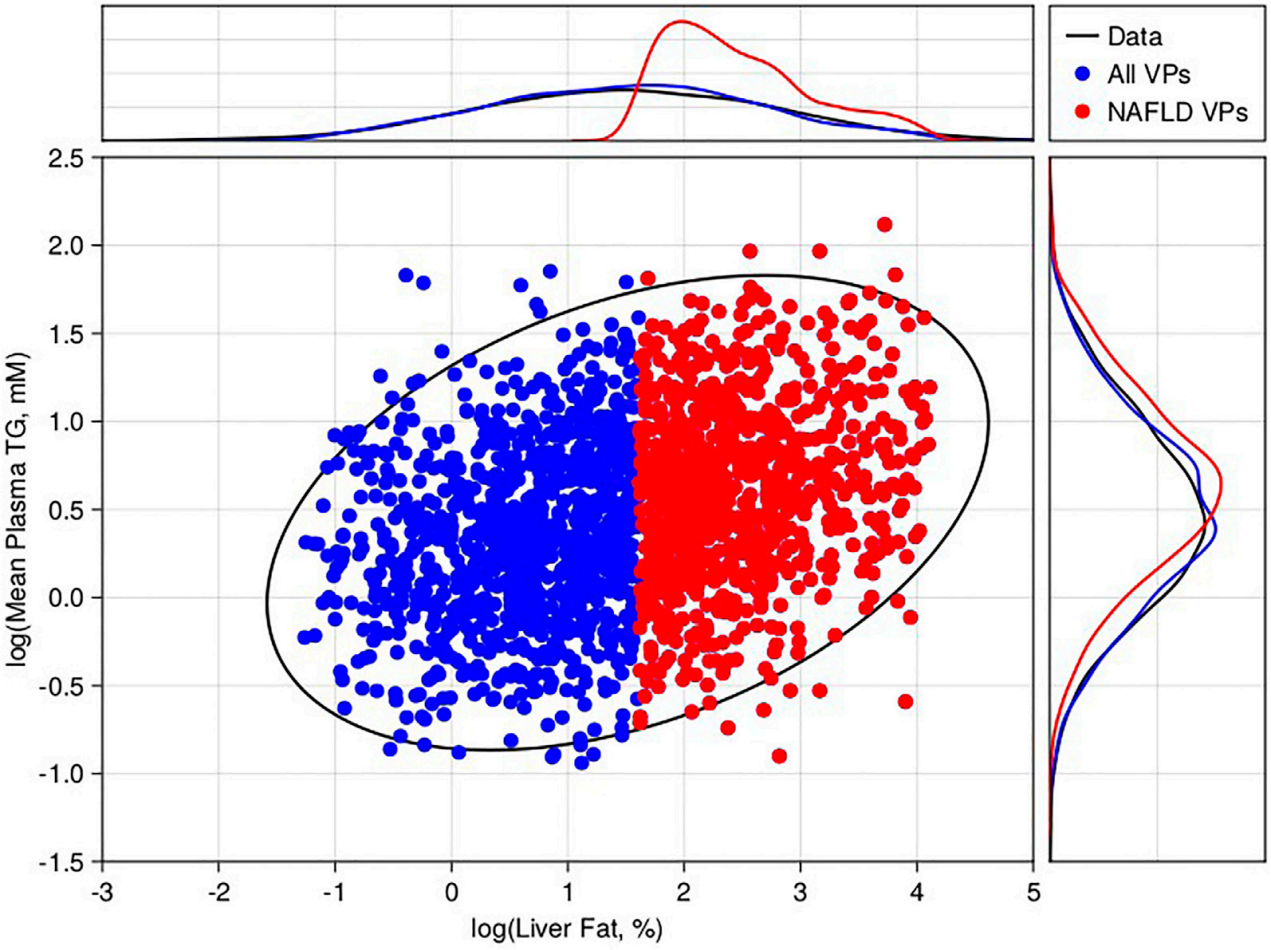
Virtual population selection with comparison to literature-derived constraints. 2D scatter compares the steady state 24-h average plasma triglycerides versus liver fat % for 2,000 selected virtual patients (blue—non-NAFLD, red—NAFLD). For comparison, we calculated the 95% confidence interval of the 2D-lognormal distribution derived from literature (black ellipse). The marginal distributions for the virtual patients (blue and red lines) for liver fat % (top) and plasma triglycerides (top) are also shown versus the data (black lines).

Following the methodology of Section 2.4, we generated 500,000 plausible patients by drawing alternative parameters using a Metropolis-Hastings algorithm, simulating to steady state, and checking the final steady state was within the pre-defined state limits. From the initial plausible population, a final virtual population of 1,900 virtual patients was selected (Figure 2), including 900 virtual patients with baseline liver fat > 5% (NAFLD virtual patients). This cohort of NAFLD virtual patients was used to test the model’s response to interventions.

### 3.2 Model Validation With Pioglitazone and Diet Intervention.

**FIGURE 3:**
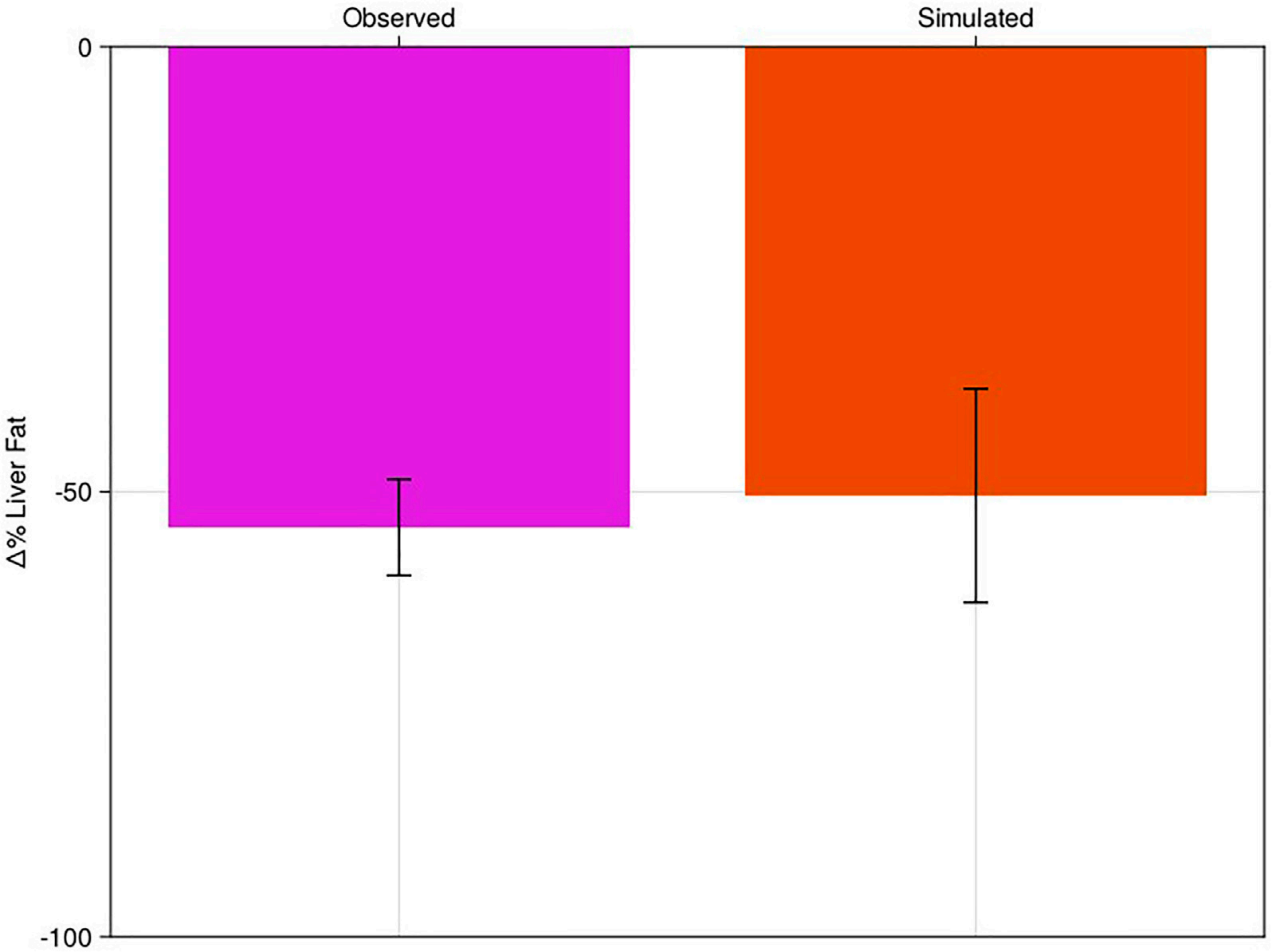
Percent change in liver fat of virtual population to pioglitazone-like therapy with comparison to literature. Clinical results of Belfort et al. (pink bar) compared to the NAFLD virtual patient response to pioglitazone (red). Bars are mean response of the cohort or virtual patients; error bars are estimated standard deviations.

Pioglitazone is a peroxisome proliferators-activated receptor gamma (PPAR) agonist and putative insulin sensitizer approved for the treatment of type 2 diabetes due to its efficacy on reducing plasma glucose (Phatak and Yin 2006). Due to its mechanism of action, pioglitazone has also been investigated for the treatment of NAFLD (Belfort et al., 2006; Gastaldelli et al., 2021). Following the methods of Section 2.5 we simulated pioglitazone as a validation of the model’s response to intervention. We fixed the Virtual Population created in Section 3.1 with no new fitting to the trial data. We induced a step change of −28% in the NEFA uptake to the model to simulate the estimated observed mean reduction in NEFAs in the Haufe et al. (2013) study. We then simulated the model for 24 weeks, allowing it to equilibrate at a new liver fat level for each patient and compared the results to the published cohort-level data from Belfort et al. (Figure 3; Supplementary Figure S1A).

**FIGURE 4:**
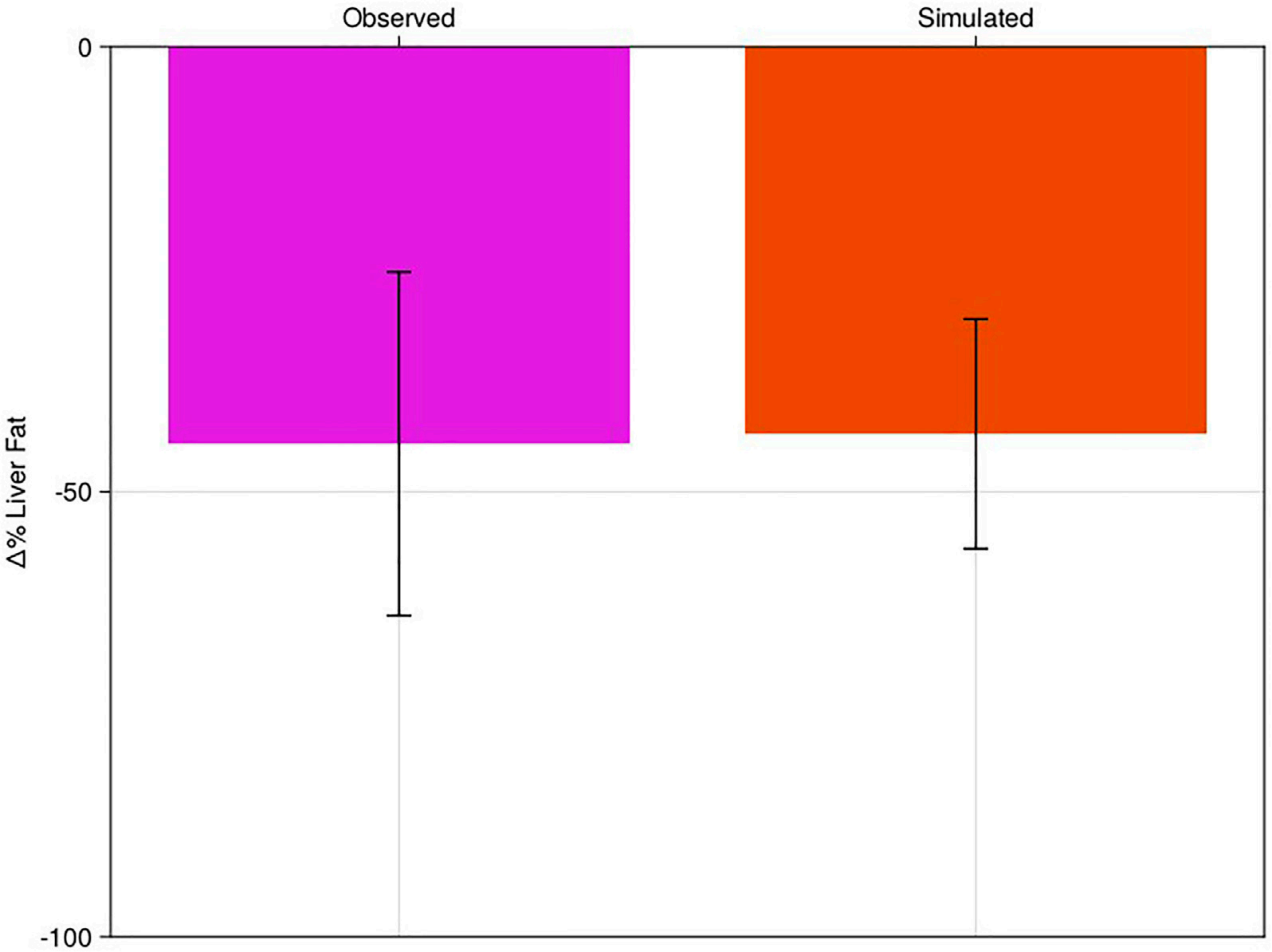
Percent change of liver fat of virtual population to diet-like therapy with comparison to literature. Clinical results of Haufe et al. (pink bar) compared to the NAFLD virtual patient response to dietary intervention (red). Bars are mean response of the cohort or virtual patients; error bars are estimated standard deviations.

Simulation of dietary intervention was like pioglitazone therapy, but the “pharmacodynamics” of the intervention are more complicated, allowing us to test additional sensitivities of the model (Section 2.6). Like the pioglitazone intervention, we started with the NAFLD cohort established in Section 3.1 and did no additional fitting based on the dietary literature results. We simulated the model for 26 weeks and compared the % change in liver fat to the published values from Haufe et al. (2013) (Figure 4; Supplementary Figure S1B).

Based on our assessment of a reasonable mean response of the model, relative to clinical variability, we felt confident that the base model parameterization was reasonable to assess against novel interventions.

### 3.3 Sensitivity Analysis

**FIGURE 5:**
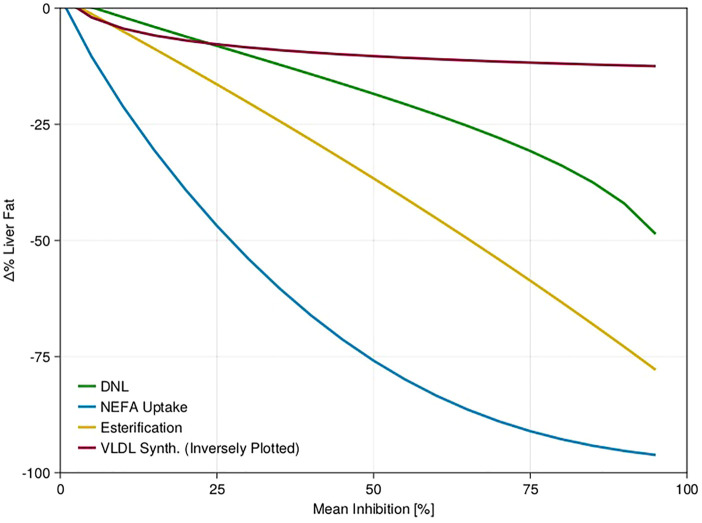
Sensitivity analysis of mean NAFLD virtual population response to step changes in five model fluxes. Fluxes were varied as described in the text for four different fluxes. Three fluxes were inhibited, one flux (VLDL synthesis) was activated. For the activated flux we plotted it on the same scale as the other three by plotting the x-axis inversely. For clarity, only the mean response of the NAFLD virtual patients is shown.

Having established the model, at least at steady state, we wished to use the model as a tool to help us understand how future treatments for NAFLD may translate from preclinical to clinical efficacy. Dietary intervention and pioglitazone treatment demonstrated there are many ways to reduce liver fat, which may not all be equal for efficacy. We performed a sensitivity analysis on four points of intervention assuming a constant 24-h level of inhibition or activation for 26 weeks (Section 2.7). We observed that the model is most sensitive to NEFA uptake to the hepatocytes, followed by DNL and esterification, and least sensitive to VLDL activation (Figure 5; Supplementary Figure S2).

## 4 Discussion

NAFLD is a complex disease whose etiology is poorly understood. We do not yet know the best point(s) of intervention and it is seeming unlikely that any one therapy will be the “silver bullet” for all forms of the disease. Therefore, the medical community will likely be attacking treatment of NAFLD/NASH from several angles: inflammation, fibrosis, and metabolic. The model presented here provides a starting point for understanding metabolic treatments.

The sensitivity analysis we performed provides a high-level indicator of the likelihood of success of a general therapeutic strategy. With a goal of reducing liver steatosis, the results of our simulation studies suggest that the most effective therapeutic strategy is to reduce the carbon flux *via* NEFAs from the adipose tissue to the liver. This observation agrees well with positive clinical trials for pioglitazone. Treatment of NAFLD patients with GLP-1 agonists, putatively acting through both anorexic effects and insulinotropic effects to suppress lipolysis, seems to support this prediction as well (Newsome et al., 2021). Direct inhibition of lipogenic flux in the liver, *via* DNL or esterification suppression, also was found to be a potent mechanism for reducing steatosis. With multiple viable avenues, the best therapeutic strategy may come down to the practical druggability of targets or a combination of different approaches.

Any model is a necessary simplification of the true biology; however, the goal is to create a model that is still fit-for-purpose. Here we presented a base model that can be expanded as required for specific research questions. As part of our model-building, we made the explicit assumptions to use coarser (24-h average) dynamics for the metabolic pools of the model. While potentially limiting for certain research questions (e.g., dose timing relative to a meal), this decision keeps the model dynamics on a timescale with the vast majority of published clinical data to date. In addition to allowing the model to be reasonably constrained by the available data, an added benefit is coarser dynamics will have a significant numerical performance benefit for practical simulation of the model. Our assumption that a 24-h averaged model can still be of practical benefit is justified by the model’s ability to reproduce liver fat changes for complex interventions, like diet or pioglitazone, without refitting a virtual population generated based on the general literature.

While the model showed a close quantitative match to the mean percent change in liver fat of the two intervention studies, it is worth noting that quantitatively capturing the clinical variability of any given study can be very difficult. Simulating the variability likely requires a specialized virtual population (subselected or specially generated) for the cohorts of that study and a careful assessment of variability of treatment (i.e., pharmacokinetics, adherence) and possibly interoccasion variability. One other limitation of the current validation studies are the pleiotropic effects of both pioglitazone and diet. For both we chose to focus on some of the best documented or hypothesized metabolic effects, but others are possible. Primarily this meant the effect would be driven through reductions of NEFAs and DNL. In the case of pioglitazone, PPARγ is expressed in both the liver and macrophages of rodents, but to a lower level than observed in adipose (Czaja 2009). However, we cannot necessarily discount the presence of PPARγ in these extra-adipose tissues contributes to NAFLD outcomes beyond steatosis (e.g., inflammation). While the approach taken here captured the steatosis endpoint reasonably, a more thorough understanding of pioglitazone’s clinical efficacy should be systematically undertaken to understand treatment of the disease more holistically.

A few areas of potential interest for future expansion of the model include regulation of lipogenic genes by SREBP1c, hourly dynamics of DNL and lipolysis from adipose in response to food intake, and more detailed handling of lipoprotein dynamics in the plasma. Beyond these metabolism-centric additions, we could also consider the lifecycle of the hepatocyte and how it is affected by lipotoxicity. Incorporating the lifecycle of the hepatocyte and turnover will also allow the direct use of commonly collected biomarkers of liver function, like alanine aminotransferase (ALT) and aspartate aminotransferase (AST). Ultimately, considering hepatocyte apoptosis and necrosis in response to stress could lead to an evaluation of how to couple the metabolic disorder of NAFLD with the inflammatory response of NASH and consider the progression/reversal of the disease. However, each of these steps should be taken as required for a particular drug-discovery research question of translation or extrapolation from short-term to longer-term trials.

The model we presented is the basis of a flexible QSP platform for understanding metabolic therapies, easily expanded to include new targets, and amenable to exploring potential combination therapy approaches. Based on currently available clinical and biological data, any mechanistic model of NAFLD pathophysiology will necessarily require a detailed and thorough exploration of uncertainty. The advantage of the model we presented here is its size and speed, which allows for development and simulations of large virtual populations. This flexibility is a crucial facilitator for providing timely and robust simulations for clinical applications. By applying this model to existing and emerging clinical and biological data it will provide further quantitative understanding of the human pathophysiology of NAFLD and how to best design treatments for the early phases of the disease.

## Data Availability

The original contributions presented in the study are included in the article/Supplementary Material, further inquiries can be directed to the corresponding author.
