# Supplementary material for: A Quantitative Systems Pharmacology Model of Liver Lipid Metabolism for Investigation of Non-Alcoholic Fatty Liver Disease
Source: Front Pharmacol. 2022 Jul 19;13:910789. doi: 10.3389/fphar.2022.910789 (PMC9343875; doi:10.3389/fphar.2022.910789)
Supplement: Supplementary file 1 [file DataSheet1.docx]

Supplementary Material For: A Quantitative Systems Pharmacology Model of Liver Lipid Metabolism for Investigation of Non-Alcoholic Fatty Liver Disease

Theodore R. Rieger^1*^, Richard J. Allen^1^, C.J. Musante^1^

^1^Quantitative Systems Pharmacology, Early Clinical Development, Pfizer Inc

# Fitted parameters and baseline values

Table S1. Summary of key parameters for the model. Model symbol references to the source code usage.

| **Description** | **Model Symbol** | **Units** | **Basal Value*** | **Calibration** |
| --- | --- | --- | --- | --- |
| TG clearance by plasma lipases (excluding hepatic) | klipase_clear | 1/day | 1.7E+01 | Mass balance from typical TG concentrations and appearance rates due to VLDL + Chylomicrons |
| Sensitivity (exponent) of NEFA uptake in liver to cytosolic NEFA concentrations | sens_nefa_uptake | dimensionless | 1.0E-03 | Free parameter |
| Sensitivity (exponent) of DNL changes in response to changes in beta oxidation | sens_betaox_dnl | dimensionless | 1.0E-01 | Free parameter |
| Rate of uptake of fatty acids from cytosol into the ER | kuptake_er | 1/day | 9.3E+02 | Mass balance on the ER (VLDL flux out must balance) |
| Clearance of TG from plasma into liver | kuptake_liver_tg | 1/day | 5.7E-01 | Back-calculated based on fractional contributions in (Lambert, Ramos-Roman et al. 2014) |
| Rate constant of synthesis of TG in the cytosol (3 FA --> TG) | ksynth_cy_tg | 1/(mM^2*day) | 1.4E+04 | Mass balance with lipolysis rate |
| Rate constant of lipolysis of TG in the cytosol (1 TG --> 3FA) | klipo_cy_tg | 1/day | 2.8E-01 | Based on the flux of VLDL export from the liver and contributions from different sources in (Fabbrini, Mohammed et al. 2008) |
| Rate constant of synthesis of TG in the ER (3 FA --> TG) | ksynth_er_tg | 1/(mM^2*day) | 1.3E+05 | Assumed equal to cytosolic rate |
| Rate constant of beta oxidation of fatty acids by the mitochondria | kbetaox | 1/day | 1.7E+03 | Based on liver BMR and assumption that fat is the primary contributor to liver oxidation. |
| Maximum rate of VLDL export from the liver to plasma | emax_vldl_prod | mmols-TG/day | 3.4E+01 | Based on (Adiels, Taskinen et al. 2006) |
| Half-maximal concentration for liver fat on VLDL production | ec50_vldl_prod | mM | 2.7E+01 | Based on (Adiels, Taskinen et al. 2006) |
| Absorption flux of chylomicron into plasma per day | chylo_basal_flux | mmols-TG/day | 9.8E+01 | Calculated based on western diet and bioavailability of fat. |
| Synthesis flux of de novo synthesized fatty acids in liver | dnl_basal_flux | mmols/day | 9.7E+00 | Back-calculated based on fractional contributions in (Lambert, Ramos-Roman et al. 2014) |
| Uptake flux for NEFAs from plasma to cytosol | nefa_uptake_flux | mmols/day | 1.7E+02 | Back-calculated based on fractional contributions in (Lambert, Ramos-Roman et al. 2014) |

*Algebraic calculation of basal values for all parameters and initial states can be found in supplemental *Pluto* notebook: *derived_parameters.jl*

# Pioglitazone calculations

The percentage reduction of non-esterified fatty acids (NEFAs) was calculated as:

Δ%NEFA = Δ %NEFA-Pio – Δ %NEFA-Placebo

With:

Δ %NEFA-Pio = (weight OGTT)* Δ %NEFA-Pio-OGTT + (1-weight OGTT)* Δ%NEFA-Pio-Fasting

And similar for placebo. Weight OGTT was set to 0.5.

The mean reduction of liver fat was taken directly from the text of Belfort et al. The standard deviation of the percent change was estimated from Figure 1C with an *assumed* correlation coefficient of rho = 0.9 pre-/post- treatment via simulation using a joint log-normal distribution.

# Diet calculations

Liver fat and adipose mass values (visceral + subcutaneous) were taken from Haufe et al. 2013, Table 2, NAFLD group, 6-month/Baseline (no placebo group).

For liver fat, the standard deviation of the percent change was calculated from the values in Table 2 with an *assumed* correlation coefficient of rho = 0.9 pre-/post- treatment via simulation using a joint log-normal distribution for liver fat.

The fractional reduction in de novo lipogenesis (DNL) was calculated from Schwartz et al. 1995, Figure 5. The percent change from eucaloric to -25% calories was calculated for both fed and fasted and then averaged (like pioglitazone, the weight was 0.5 for each condition). The value at -25% was then linearly scaled to -20% for the calculated food intake reduction in Haufe et al.

# Virtual population response to pioglitazone and diet


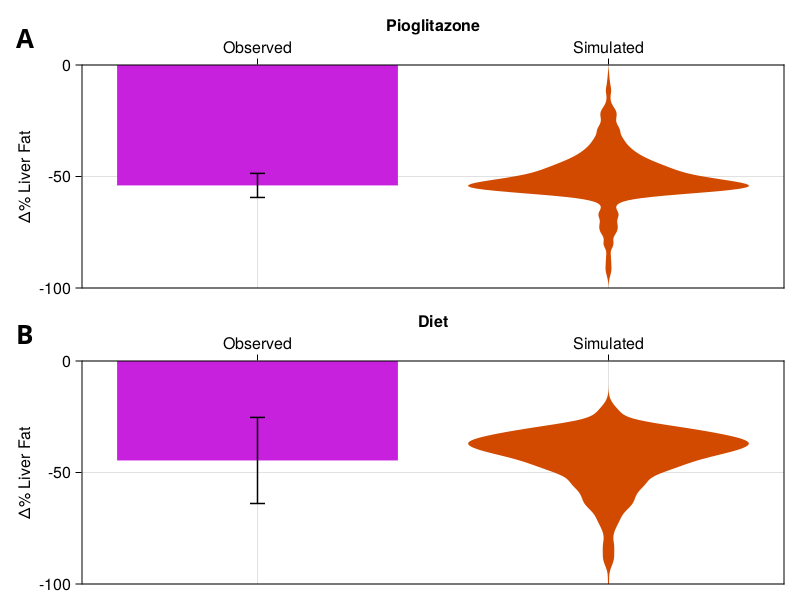


Figure S1. Pioglitazone (A) and diet (B) simulations from main text with distribution of virtual patients. Observed (pink bars) is as before from text. Simulations are the distribution of the virtual population in response to either stimulus.

# Virtual population response for sensitivity analysis


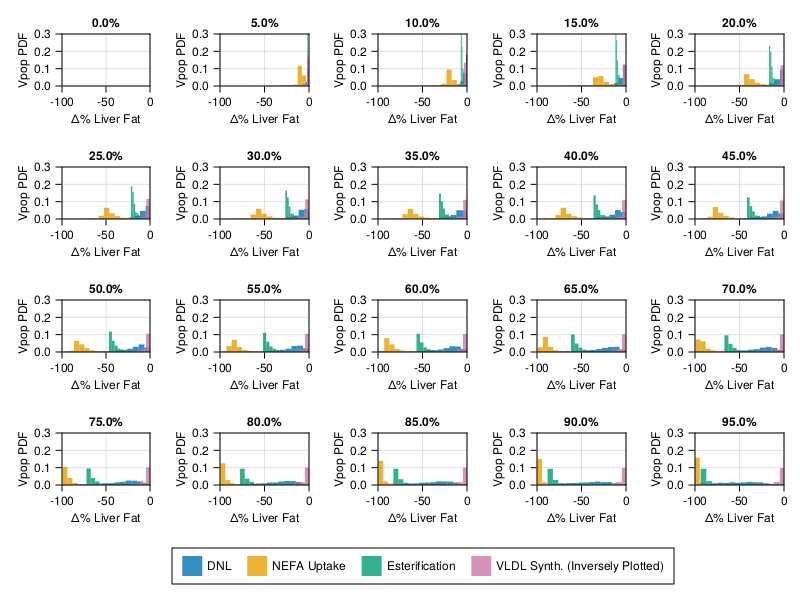


Figure S2. Sensitivity analysis from Figure 5 with full virtual population plotted as histograms. Each panel is the percent inhibition (or activation) for the four different points of potential intervention.

# Supplementary references

Adiels, M., M. R. Taskinen, C. Packard, M. J. Caslake, A. Soro-Paavonen, J. Westerbacka, S. Vehkavaara, A. Hakkinen, S. O. Olofsson, H. Yki-Jarvinen and J. Boren (2006). "Overproduction of large VLDL particles is driven by increased liver fat content in man." Diabetologia **49**(4): 755-765.

Fabbrini, E., B. S. Mohammed, F. Magkos, K. M. Korenblat, B. W. Patterson and S. Klein (2008). "Alterations in adipose tissue and hepatic lipid kinetics in obese men and women with nonalcoholic fatty liver disease." Gastroenterology **134**(2): 424-431.

Lambert, J. E., M. A. Ramos-Roman, J. D. Browning and E. J. Parks (2014). "Increased de novo lipogenesis is a distinct characteristic of individuals with nonalcoholic fatty liver disease." Gastroenterology **146**(3): 726-735.
